# Ticks, *Ixodes scapularis*, Feed Repeatedly on White-Footed Mice despite Strong Inflammatory Response: An Expanding Paradigm for Understanding Tick–Host Interactions

**DOI:** 10.3389/fimmu.2017.01784

**Published:** 2017-12-18

**Authors:** Jennifer M. Anderson, Ian N. Moore, Bianca M. Nagata, José M. C. Ribeiro, Jesus G. Valenzuela, Daniel E. Sonenshine

**Affiliations:** ^1^Vector Biology Section, Laboratory of Malaria and Vector Research, National Institute of Allergy and Infectious Diseases (NIH), Rockville, MD, United States; ^2^Infectious Disease and Pathogenesis Section, Comparative Medicine Branch, National Institute of Allergy and Infectious Diseases (NIH), Rockville, MD, United States; ^3^Vector Molecular Biology Section, Laboratory of Malaria and Vector Research, National Institute of Allergy and Infectious Diseases (NIH), Rockville, MD, United States; ^4^Department of Biological Sciences, Old Dominion University, Norfolk, VA, United States

**Keywords:** *Ixodes scapularis*, *Peromyscus leucopus*, inflammation, T-cells, macrophages, sessile

## Abstract

Ticks transmit infectious agents including bacteria, viruses and protozoa. However, their transmission may be compromised by host resistance to repeated tick feeding. Increasing host resistance to repeated tick bites is well known in laboratory animals, including intense inflammation at the bite sites. However, it is not known whether this also occurs in wild rodents such as white-footed mice, *Peromyscus leucopus*, and other wildlife, or if it occurs at all. According to the “host immune incompetence” hypothesis, if these mice do not have a strong inflammatory response, they would not reject repeated tick bites by *Ixodes scapularis*. To test this hypothesis, histopathological studies were done comparing dermal inflammation in *P. leucopus* versus guinea pigs, *Cavia porcellus*, repeatedly infested with *I. scapularis*. In *P. leucopus*, the immune cell composition was like that seen in laboratory mouse models, with some differences. However, there was a broad sessile lesion with intact dermal architecture, likely enabling the ticks to continue feeding unimpeded. In contrast, in *C. porcellus*, there was a relatively similar mixed cellular profile, but there also was a large, leukocyte-filled cavitary lesion and scab-like hyperkeratotic changes to the epidermal layer, along with itching and apparent pain. Ticks attached to sensitized *C. porcellus* fed poorly or were dislodged, presumably due to the weakened anchoring of the tick’s mouthparts cemented in the heavily inflamed and disintegrating dermal tissues. This is the first time that the architecture of the skin lesions has been recognized as a major factor in understanding tick–host tolerance versus tick bite rejection. These findings broadly strengthen previous work done on lab animal models but also help explain why *I. scapularis* can repeatedly parasitize white-footed mice, supporting the “immune evasion theory” but cannot repeatedly parasitize other, non-permissive hosts such as guinea pigs.

## Introduction

Ticks are hematophagous arthropods that can transmit a variety of infectious agents including bacteria, viruses, and protozoa (1). Following its attachment, the tick secretes numerous pharmacologically active compounds into the host skin that helps it to remain attached, undetected, and to engorge with blood (2). The most important species in the United States is *Ixodes scapularis*, the primary vector for Lyme borreliosis, human granulocytic anaplasmosis, human babesiosis, and other disease-causing agents (3).

Resistance to a tick bite is expressed by many vertebrate species, although exceptions occur. Ticks, like other blood-feeding ectoparasites, stimulate a broad spectrum of host immune responses that impairs their ability to feed, disrupt their development, and ultimately kill them (4). Consequently, ticks have evolved a highly sophisticated array of anti-hemostatic and immunomodulatory countermeasures that modulate or even completely disable the host’s responses, especially in tick-naïve animals. Tick saliva contains a veritable pharmacopeia of bioactive molecules, with nearly 500 secreted proteins and peptides and comprising at least 25 different protein families (3, 5, 6). Collectively, these salivary agents prevent blood coagulation; inhibit immunoglobulins; disrupt or compromise the host complement system; and alter cytokine mediated signal transduction. These diverse salivary molecules have vasodilator, antiplatelet, anti-hemostatic, anticoagulant, anti-histamine, and immuno-suppressor functions (6). Occasionally, multiple strategies of immune evasion have arisen within a single tick species, e.g., *I. scapularis* (7).

Despite the tick’s impressive repertoire of anti-hemostatic agents, many animals mount a vigorous immune response, especially after repeated tick-feeding attempts. This was first reported by Trager (8) for guinea pigs (*Cavia porcellus*) infested with *Dermacentor variabilis*. This phenomenon is known as acquired resistance to tick bites (4) and is strongly dependent on the nature of the innate and adaptive host immune responses. However, the laboratory mouse is an exception to this common host response. Those mice do not become resistant or, in some cases, develop only limited inflammatory responses to repeated tick bites, neither of which leads to tick rejection (9, 10). Laboratory mice were shown to be tolerant to repeated infestations by *I. scapularis* (11), but whether white-footed mice (*Peromyscus leucopus*) would mount any immune response to *I. scapularis* has not been previously investigated.

The histopathology of tick-bite rejection has been the subject of intense study for many decades (4, 9). The antihemostatic repertoire of tick salivary proteins and peptides induces vascular dilation to enhance blood flow and prevent blood coagulation or wound healing. Although numerous macrophages, neutrophils, and lymphocytes enter the wound site, few eosinophils are attracted to the feeding lesion and the tick can continue engorging on the accumulating blood pool without resistance from its host. Damage to dermal capillaries, venules, and other blood vessels allows blood to pool around the tick’s mouthparts. However, in guinea pigs, an abnormal host for most tick species, the histopathology of the feeding lesion following a second infestation by the same tick species reveals a very different picture. Numerous inflammatory cells, i.e., macrophages, neutrophils, eosinophils, and lymphocytes accumulate around the tick’s mouthparts, blocking blood uptake and minimizing the tick’s ability to engorge (4). Ticks attempting to feed again on these now tick-immune hosts encounter an even more vigorous rejection; strongly upregulated pain and itch responses induce the host to dislodge or kill them. Exceptions to this acquired resistance phenomenon occur; e.g., mice (*Mus musculus*) subjected to repeated infestations with *I. scapularis* do not reject the feeding ticks even though they may develop an increasingly prominent inflammatory response (10–12). In contrast, guinea pigs, as noted above, strongly resist further tick challenges following even one prior tick infestation (13). Experience with tick feeding on rabbits (*Oryctolagus cuniculus*) also showed very strong anti-tick rejection by the second or third conspecific tick challenge [(14) Sonenshine unpublished data].

It is often assumed that *P. leucopus* do not reject *I. scapularis* because these animals fail to express an increasingly strong and acute dermal inflammatory response to repeated tick bite challenges, the so-called “host immune incompetence” hypothesis. Alternatively, the absence of tick rejection could derive from the tick’s “immune evasion” of the host’s immune response (15). Therefore, although many of the familiar histopathological inflammatory features may appear in the skin during tick feeding, it is likely that the immunological “memory,” i.e., adaptive resistance, is disabled (i.e., immune evasion). Consequently, it was expected that there would be little change in the histopathological presentation of host skin during subsequent tick challenges, thereby allowing tick larvae and/or nymphs to feed successfully.

To determine whether these hypotheses are valid, we conducted histopathological and immunohistochemical (IHC) studies of *P. leucopus* skin tissues (including attached ticks) during successive tick feeding challenges. For a control, we conducted comparable histopathological and IHC studies of the skin of tick-infested guinea pigs (*C. porcellus)*, a species known to exhibit strong anti-tick rejection responses, for comparison with the mouse studies.

Here, we report results that add to and strengthen the existing literature on immune responses to tick bites on the reservoir and non-reservoir hosts. Our results improve our understanding of why *I. scapularis* repeatedly parasitizes white-footed mice, a relatively “permissive” host, but not other, non-permissive hosts such as guinea pigs. These findings support the “tick immune evasion” theory to explain the lack of rejection responses when these mice are bitten by *I. scapularis* nymphs.

## Materials and Methods

### Ticks and Experimental Animals

Pathogen-free nymphal *I. scapularis* ticks were obtained from colonies maintained at the Oklahoma State University (Stillwater, OK, USA). Ticks were maintained in an incubator at 24°C and 90% relative humidity under a 14:10 h photoperiod at the Laboratory for Malaria and Vector Research (LMVR), NIAID, NIH, Rockville, MD, USA. 3- to 6-month-old female white-footed mice, *P. leucopus*, LL stock, were obtained from the University of South Carolina *Peromyscus* Genetic Stock Center (Columbia, SC, USA). The *P. leucopus* LL stock was derived from 38 wild mice captured between 1982 and 1985. Approximately 3-week-old (250–300 g) pathogen-free out-bred female albino Hartley guinea pigs (Crl: HA), *C. porcellus*, were obtained from Charles River Laboratories (Wilmington, MA, USA). White-footed mice and guinea pigs were maintained as approved by the National Institutes of Health Animal Care and Use Committee protocol (ASP LMVR6).

### Tick Exposure to White-Footed Mice and Guinea Pigs

A total of 17 mice, *P. leucopus* and 8 guinea pigs, *C. porcellus*, were used in this study. Prior to tick placement, the mice were weighed, sedated (100 mg/kg of ketamine and 10 mg/kg of xylazine; IM), and 10 nymphal ticks were placed on the head between the ears. Similarly, guinea pigs were sedated (50 mg/kg ketamine and 5 mg/kg xylazine; IP) and the area between the ears was shaved. Since guinea pigs are much larger than white-footed mice, 20 nymphal ticks were placed on the shaved area and allowed to attach. For the guinea pigs, each animal’s head was completely covered with stockinet during sedation until all ticks attached, which was verified by a gentle tug using forceps. Once ticks were attached, the stockinet was removed and an Elizabethan collar (Webster Veterinary Supply, Sterling, MA, USA) was placed around the neck to prevent grooming and was not removed until all ticks were removed or fed to repletion (approximately 6–8 days’ post-attachment). Animals were monitored until they were fully conscious and any ticks not attached at that time were removed. When ticks were attached, guinea pigs and mice were housed individually in specialized triple containment cages. Each guinea pig or mouse was placed in the innermost container, including a metal rack upon which the animal could rest. Food was provided from a metal bowl situated on the rack; water was provided by a bottle hung above the rack. The inner container was placed within a larger outer container that held about 1″ of water. The water and a layer of double-sided sticky tape around the upper edge of the outer container prevented unattached ticks from escaping. Once all ticks were accounted for, animals were returned to normal bedding. Guinea pigs and mice were exposed to ticks three successive times separated by 2-week intervals. Selected animals were removed from the study on day 0 at 2, 6, and 12 h and again on days 1–4 post attachment for each successive tick challenge to collect skin biopsies at the sites of tick attachment for histology. Remaining animals were examined daily until all ticks dropped off naturally. Excluding the guinea pigs that were sacrificed for sampling in the first tick challenge, tissue samples were collected from several of the same animals infested again during the third tick challenge at the same time intervals. Food and water was provided *ad libitum* for the animals during the entire tick feeding periods.

### Skin Biopsy Collection and Processing

At the various time points noted above, skin biopsies (2–4 mm) were collected from anesthetized animals using a sterile dermal biopsy punch. One mouse or guinea pig was used for each time point. Where possible, i.e., when two or more ticks were attached, multiple samples were collected from separate locations, e.g., ear, eyelid, or head. Following collection of the skin biopsies and while they were still sedated, the animals were immediately euthanized by either CO_2_ inhalation (mice) or pentobarbital overdose (80 mg/kg IC; guinea pigs). Biopsy samples were placed immediately in 10% formaldehyde solution. Each time point (i.e., hour or day post-challenge) and the locations (e.g., head, ear, eyelid) where the biopsy samples were taken from each animal were noted. Time points and body sites biopsied where ticks had attached for *P. leucopus* are listed in Table 1. Similar data were collected for guinea pigs but omitted from the table since tissue biopsies were done only on days 3 and 4 of the first and third tick challenges. Although animals and ticks were monitored during the second tick challenge, no data were collected since the goal of the study was to examine animals after the greatest number of sequential tick challenges.

**Table 1:** Inflammatory responses in white-footed mice (*Peromyscus leucopus*) following successive feeding challenges with nymphal ticks, *Ixodes scapularis*.

Day of examine	Skin sample number	Animal number	Location on body	Inflammation grade response^a^	Characterization inflammation and immune cell types present
**1st challenge**					

H2	P001	94	Ear	Normal—not infested	None
H2	P002	94	Head	Normal—not infested	None
H2	P038	94	Eyelid	0	None
H6	P043	67	Ear	+	Focal, superficial dermal inflammation
H6	P044	67	Eyelid	+	Slight, mixed, macrophages and neutrophils and very few eosinophils
D1	P007	8	Head	+	Very few, mixed, macrophages/neutrophils/T-cells
D1	P008	8	Base of ear	+	Very few, mixed, macrophages/neutrophils/T-cells
D1	P009	8	Base of ear	+	Mixed. Focal diffuse, broad inflammatory response, edema
D1	P010	8	Base of ear	+	Mixed, early focal inflammation
D1	P011	8	Head	+	Mixed, macrophages/neutrophils/T-cells
D2	P004	2	Near eye	+	Mixed, macrophages/neutrophils/T-cells
D2	P005	2	Near eye	+	Mixed macrophages/neutrophils/few eosinophils
D2	P012	N/A	Ear	0	Normal ear sample; no tick attached
D2	P019	99	Head	+	Mixed macrophages/neutrophils/few eosinophils dermal infiltrate
D2	P020	99	Head	++	Mixed, spreading inflammation, hyperemic vessels
D2	P023	99	Eye	++	Mixed, macrophages/neutrophils/few eosinophils, focal hyperplasia, parakeratotic hyperkeratosis with serocellular crusting
D3	P006	1	Behind eye	++	Mixed, focal diffuse intense macrophagic/neutrophilic/inflammation
D4	P013	3	Head	+++	Mixed, with many macrophages/neutrophils and few eosinophils, focal diffuse intense inflammation, hemorrhage
D4	P014	3	Head	+++	Mixed, subacute moderate diffuse dermatitis, mostly macrophages and neutrophils, focal hyperplasia, hyperkeratosis around tick bite

**3rd challenge**					

H2	P040	78	Base of ear	0	None
H2	P041	78	Eyelid	0	None
H2	P042	78	Eyelid	0	None
H6	P045	74	Head	0	None
H6	P046	74	Eyelid	0	None
H6	P047	74	Eyelid	+	Mixed T-cells macrophages
H6	P048	74	Ear	+	Mixed, macrophages, neutrophils, few eosinophils
H12	P050	66	Ear	++	Mixed, macrophages, neutrophils, few eosinophils
H12	P051	66	Eye	+++	Mixed, many macrophages, neutrophils, few eosinophils
H12	P052	66	Eye	0	None
D1	P024	1	Ear	+++	Mixed, macrophages, neutrophils, few eosinophils
D1	P025	1	Head	+++	Mixed, macrophage/neutrophil and histiocytic inflammation extensive hyperplasia
D1	P053	65	Head	+++	Mixed, many macrophages, neutrophils, some T-cells few eosinophils
D1	P054	65	Inside eyelid	+++	Mixed, many macrophages, neutrophils, some T-cells few eosinophils
D1	P055	65	Chin	+++	Mixed, many macrophages, neutrophils, some T-cells, few eosinophils
D2	P028	2	Ear	+++	Mixed, many macrophages, neutrophils, some T-cells, few eosinophils
D2	P029	2	Head	++	Mixed, many macrophages, neutrophils, few eosinophils, widespread, extensive hyperplasia
D2	P030	2	Eye	++	Mixed, many macrophages, neutrophils, few eosinophils
D2	P031	2	Eye	++	Mixed, many macrophages, neutrophils, few eosinophils, widespread, extensive hyperplasia, ruptured blood vessels
D2	P032	2	Head	++++	Mixed, intensive macrophagic/neutrophilia, few eosinophils, very widespread inflammation, hyperplasia, hyperkeratosis
D2	P056	77	Head	+++	Mixed, many macrophages, neutrophils, few eosinophils
D2	P057	77	Chin	+++	Mixed, many macrophages, neutrophils, few eosinophils
D2	P058	77	Eye	+++	Mixed, many macrophages, neutrophils, T-cells, few eosinophils
D2	P059	77	Eye	+++	Mixed, many macrophages, neutrophils, T-cells, few eosinophils
D3	P033	3	Ear	++	Mixed, many macrophages, neutrophils, few eosinophils, necrotic skin
D3	P034	3	Base of head	+++	Mixed, many macrophages, neutrophils, few eosinophils, widespread, extensive hyperplasia, hyperkeratosis
D3	P035	3	Eyelid	++	Mixed, many macrophages, neutrophils, some T-cells, few eosinophils
D3	P036	3	Eyelid	++	Mixed, many macrophages, neutrophils, few eosinophils, hyperplasia
D3	P037	3	Head	+++	Mixed, very intensive macrophagic/neutrophilic inflammation, sessile, hyperplasia and hyperkeratosis in zone of tick bite
D4	P060	71	Eye	++++	Mixed, many macrophages, segmenters, many neutrophils, few eosinophils
D4	P061	71	Ear	++++	Mixed, many macrophages, neutrophils, T-cells, many few eosinophils
D4	P062	71	Ear	+++	Mixed, many macrophages, neutrophils, few eosinophils
D4	P063	70	Ear	++++	Mixed, macrophages, neutrophils, T-cells, few eosinophils
D4	P064	70	Eye/head	++++	Mixed, macrophages, neutrophils, T-cells, few eosinophils
D4	P065	70	Ear	+++	Mixed, macrophages, neutrophils, T-cells, few eosinophils, atypical macrophages

*^a^Key to symbols: D, day; H, hour; 0, no inflammation; +, minimal inflammatory response; ++, mild inflammatory response; +++, moderate inflammatory response; ++++, extremely strong inflammatory response*.

### Histological Procedures

Tissues from skin biopsies were formalin-fixed (10% neutral buffered formalin), embedded in histological grade paraffin, sectioned at 5 µm, and stained with hematoxylin-eosin (H&E) for examination by light microscopy. All sections were evaluated by a board-certified veterinary pathologist. All images were taken using an Olympus BX51 microscope (Olympus Corp., Alexandria, VA, USA) and photomicrographs were taken using an Olympus DP73 camera.

### Immunohistochemistry for Identification of Inflammatory Cells

Skin sections from *P. leucopus* and from *C. porcellus* were heated to 72°C for 1 h in Bond Dewax Solution (Leica) and rehydrated with alcohol-gradated washes and 1× Bond Wash Solution (Leica). Bond Epitope Retrieval Solutions (Leica) were applied to sections and heated to 100°C for 20 min for Heat-Induced Epitope Retrieval (HIER). After exposure to peroxide block (Leica) for 30 min, tissues were incubated with several antibodies including, α-myeloperoxidase (MPO, Abcam) for neutrophils, α-Major Basic Protein [Eosinophil major basic protein (EMBP), Santa Cruz Biotechnology, Paso Robles, CA, USA] for eosinophils, α-CD3 (AbD Serotec) for T-lymphocytes, α-IBA1 (ionized calcium binding adaptor molecule 1; Wako Chemicals, USA) for macrophages, and mMCP-8 (mouse-specific) for basophils (*P. leucopus* only). Following treatment with these primary antibodies, the tissue sections were rinsed (PBS), treated with the secondary antibody (Avidin-biotin-horseradish peroxidase), and colorized using 0.01% diaminobenzidine and 0.01% peroxide (H_2_O_2_) for 10 min and were counter-stained using hematoxylin. Some sections also were stained with Luna stain, a stain originally developed for identifying eosinophils (16).

## Results

### *P. leucopus* First Tick Challenge

**Figure 1:**
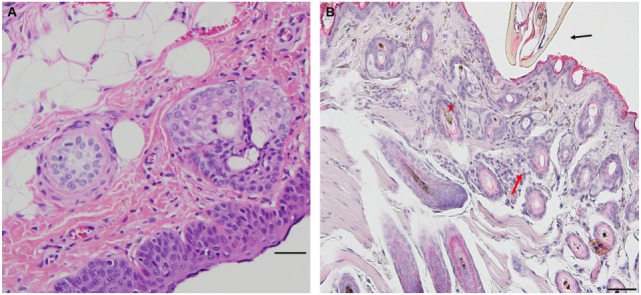
Photomicrographs showing histopathology of mouse (*Peromyscus leucopus*) skin before and after attachment of nymphal tick, *Ixodes scapularis*. First tick challenge. Stain hematoxylin and eosin (H&E). **(A)** Normal skin. Bar = 100 µM. **(B)** Skin 6 h after attachment showing accumulations of neutrophils and a few eosinophils, indicating an early focal inflammatory response. Bar = 50 µM. Black arrow indicates tick capitulum; red arrow indicates invading leukocytes; asterisk indicates hair follicle.

*Peromyscus leucopus* tissues collected from a tick-naïve mouse show the characteristics of normal epidermis and underlying dermis. Blood vessels are normal, not dilated, and there is no evidence of extravasated blood and no evidence of leukocytes infiltration (Figure 1A).

*Peromyscus leucopus* tissues examined within 2 h (2) post first tick-challenge show no evidence of an inflammatory response. However, tissue samples examined within 6 h post first tick-challenge (H6) show evidence of an early inflammatory response (Table 1, first challenge). In the developing lesion below the inserted mouthparts, one may observe a small, focal accumulation of neutrophils and a few eosinophils, suggesting an early focal inflammatory response. Otherwise, there is no further accumulation of mixed polymorphs, no hyperplasia, and no hyperkeratosis (Figure 1B).

**Figure 2:**
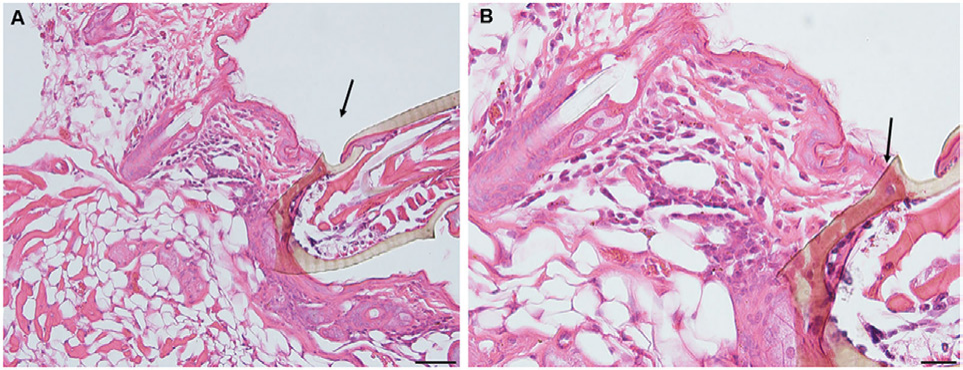
Photomicrographs showing histopathology of mouse (*Peromyscus leucopus*) skin on day 1 of first tick challenge (D1) showing an increasing inflammatory dermal response and hyperplasia. Stain H&E. **(A)** Skin with developing lesion surrounding tick mouthparts. Bar = 100 µM. **(B)** Enlargement showing detail of lesion adjacent to tick mouthparts. Bar = 50 µM. Black arrows indicate tick mouthparts.

On day 1 post-challenge, there is evidence of a superficial and diffuse mixed infiltrate indicating an inflammatory dermal response with localized hyperplasia in the developing lesion adjacent to the attached tick. Although mixed, neutrophils are the most numerous immune cells at this stage of the host reaction (Figures 2A,B).

**Figure 3:**
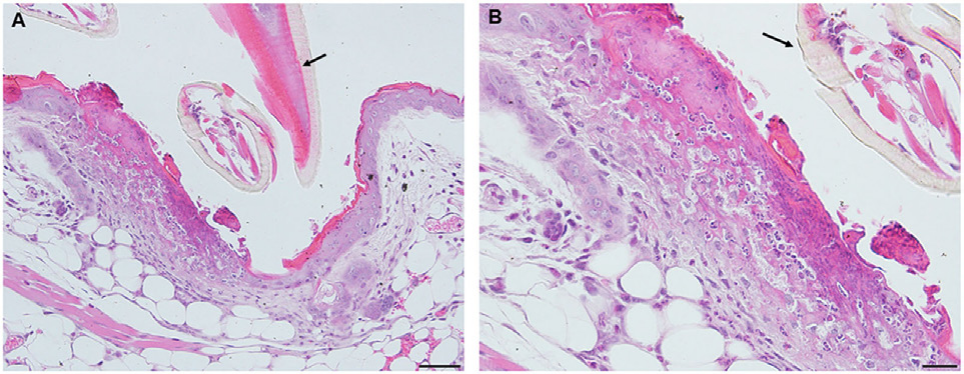
Photomicrographs showing histopathology of mouse (*Peromyscus leucopus*) skin on day 2 of first tick challenge (D2) showing an increasing inflammatory dermal response with widespread mixed leukocytic infiltrates, serocellular crusting, dermal hyperplasia, and hyperkeratosis. Stain H&E. **(A)** Skin with expanding lesion surrounding tick mouthparts. Bar = 100 µM. **(B)** Enlargement showing detail of lesion adjacent to tick mouthparts. Arrow = tick mouthparts. Bar = 50 µM. Black arrows indicate tick mouthparts.

By day 2 post first-challenge, the inflammatory response is like that seen on day 1. In addition to the superficial and diffuse mixed dermal infiltrate and focal epidermal hyperplasia, the inflammation is more widespread with serocellular crusting (i.e., serum and cells forming a crust), some extravasated blood, dilated capillaries, edema, and intravascular coagulation. Also, there is a focal region of parakeratotic hyperkeratosis (Figures 3A,B).

**Figure 4:**
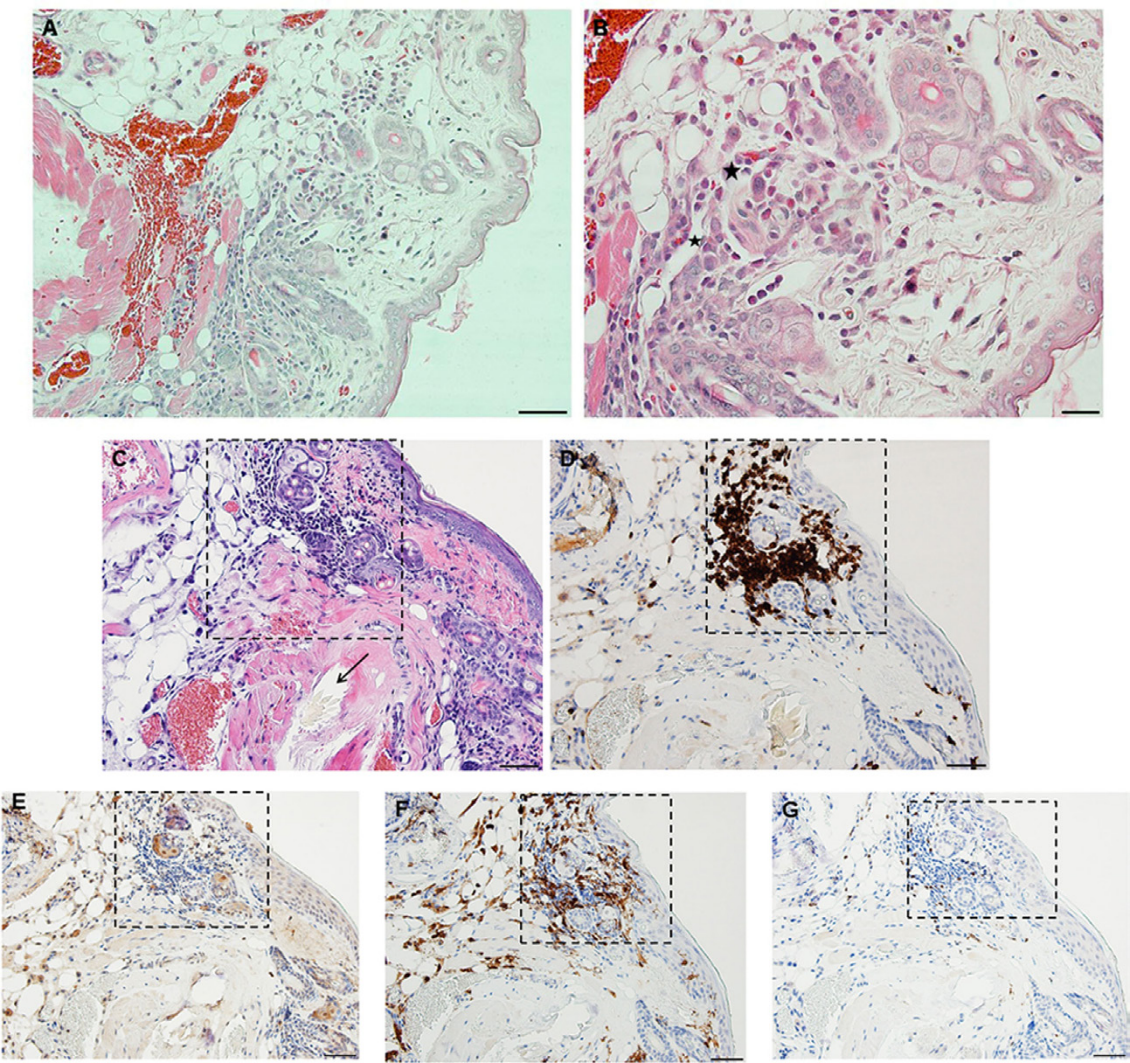
Photomicrographs showing histopathology of mouse (*Peromyscus leucopus*) skin on day 3 of first tick challenge (D3). The inflammatory response was like day 2. See text for details. **(A)** Skin area of tick-bite showing the sessile lesion, spreading out over a broad area of the tissues near the tick mouthparts, dilated blood vessels, extravasated erythrocytes, and an intense mixed but predominantly neutrophilic inflammation with some eosinophils. Stain H&E. Bar = 100 µM. **(B)** Higher magnification image showing detail of tick-bite lesion with neutrophils and few eosinophils (asterisks) invading tissues. Stain H&E. Bar = 50 µM. Photomicrographs showing histopathology of mouse (*Peromyscus leucopus*) skin on day 3 of first tick challenge (D3) showing an increasing inflammatory dermal response with widespread mixed leukocytic infiltrates, vascular disruption, and extravasated blood. **(C–G)** High magnification image showing detail of the highly focal area (box) with numerous leukocytic infiltrates adjacent to the tick-bite lesion (black arrow indicating fragments of tick hypostome). **(C)** Stain H&E. Bar = 50 µM. **(D–G)** Immunohistochemical markers for different leukocyte cell types, staining brown. **(D)** CD3 identifying numerous T-lymphocytes (dark brown) concentrated in the tick-bite lesion (box). **(E)** Eosinophil major basic protein identifying few eosinophils in or near the tick-bite lesion (box). **(F)** IBA1 identifying numerous macrophages in the tick-bite lesion (box) and surrounding dermal tissues. **(G)** Myeloperoxidase identifying few neutrophils concentrated in the tick-bite lesion (box). Bars = 50 µM.

By day 3 post first-challenge, the inflammatory response has become more intense, more hypercellular, and spreading out over a broader area of the tissues surrounding the tick-bite lesion. It is focally diffuse, with an intense mixed leukocytic inflammation, and a mild-to-moderate focal extravasation of erythrocytes. Hematoxylin and eosin (H&E) staining reveals occasional eosin-staining cells (asterisks). It also includes a background of mononuclear cells and occasional lymphocytes (Figures 4A,B). IHC staining reveals an intense, focal infiltration of T-cells and macrophages, some neutrophils but very few eosinophils (Figures 4C–G). Thus, the inflammation at this stage is primarily a T-cells and macrophage-rich dermatitis.

**Figure 5:**
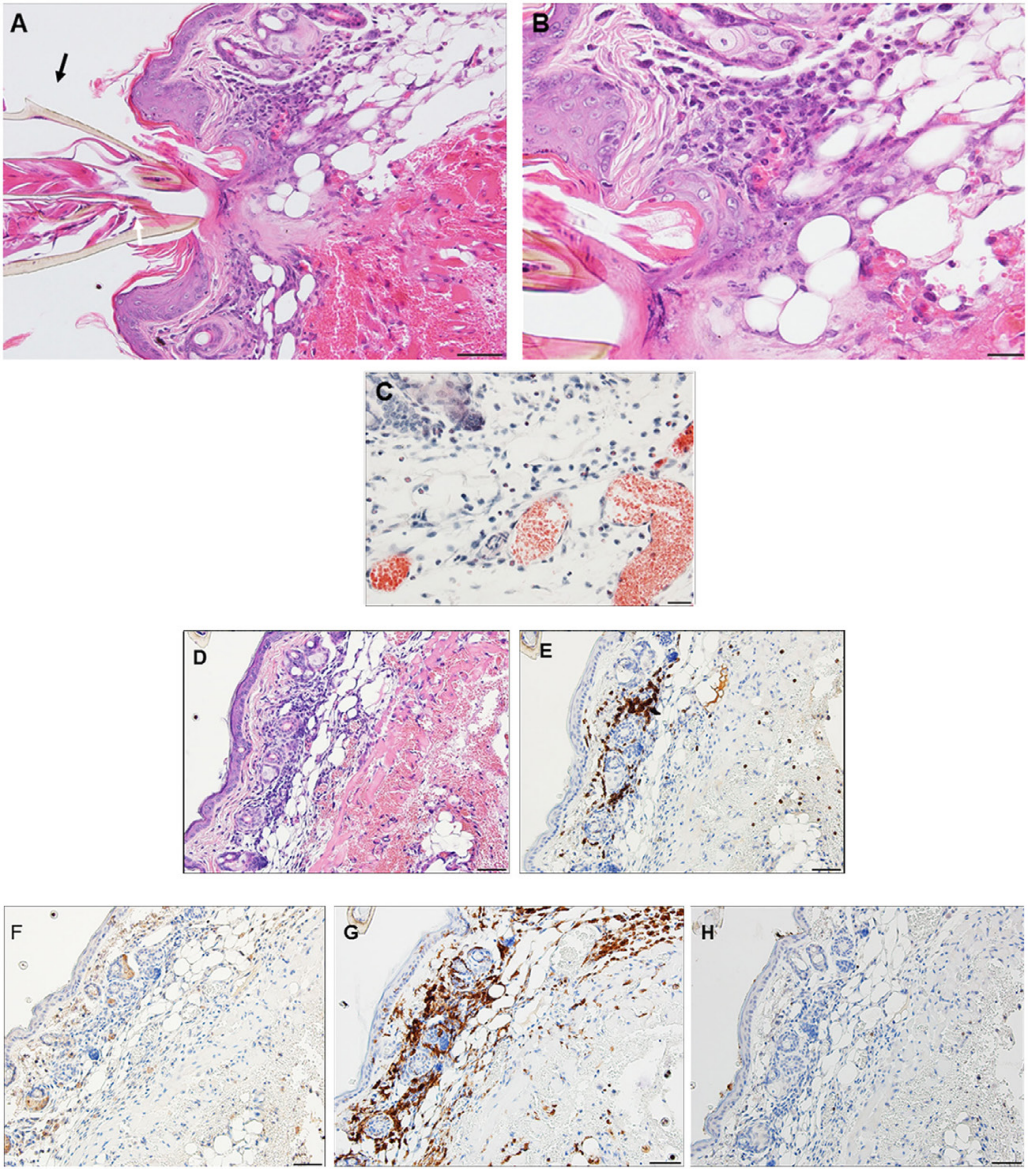
Photomicrographs showing histopathology of mouse (*Peromyscus leucopus*) skin from ear collected on day 4 of first tick challenge (D4). Acute inflammatory response with granulomatous dermatitis and hemorrhage. See text for details. **(A)** Low magnification image showing tick mouthparts, skin with tick-bite lesion, pronounced hyperplasia, and extensive hemorrhage. Stain H&E. Bar = 100 µM. **(B)** Higher magnification image showing detail of tick-bite lesion adjacent to tick mouthparts showing infusion of mixed leukocytes, including some eosinophils, macrophages, and other polymorphonuclear cell types (e.g., mast cell, black arrow). Stain H&E. Bar = 50 µM. **(C)** Detail of tick-bite lesion with Luna stain specific for eosinophils. Note greatly diluted blood vessels. Bar = 50 µM. Photomicrographs showing histopathology of mouse (*Peromyscus leucopus*) skin on day 4 of first tick challenge (D4). **(D–H)** Immunohistochemical (IHC) markers for different leukocyte cell types (brown stain) in or near the tick-bite lesion (box). **(D)** H&E stained section for comparison with IHC-stained sections following. **(E)** CD3 identifying numerous T-lymphocytes (dark brown) concentrated in the tick-bite lesion. **(F)** Eosinophil major basic protein identifying few eosinophils in or near the tick-bite lesion. **(G)** IBA1 identifying numerous macrophages in the tick-bite lesion and surrounding dermal tissues. **(H)** Myeloperoxidase; neutrophils absent from the tick-bite lesion. Bars = 50 µM.

By day 4 post first-challenge, examination of an ear sample from the region of the tick bite shows an acute inflammation, as well as focal granulomatous mixed leukocytic dermatitis with extensive hemorrhage, masses of extravasated erythrocytes, focal epidermal hyperplasia, and hyperkeratosis (Figures 5A–C). The IHC images (Figures 5D–H) show that the leukocytic infiltrates include a few eosinophils (Figure 5F) but are comprised primarily of T-cells and macrophages (Figures 5E,G). Neutrophils are rare or absent (Figure 5H). An H&E image from the identical skin section is included (Figure 5D) to facilitate comparisons with the IHC-stained sections. Focal epidermal hyperplasia and hyperkeratosis is pronounced surrounding the mouthparts of the feeding tick. The entire dermal inflammatory lesion is sessile. Despite the rapidly developing inflammation, examination of the attached tick shows a healthy body structure with no evidence of breakdown or impairment of its ability to blood feed. The tick appears to have been engorging normally.

Aside from tick specimens collected with the biopsy samples, most ticks that fed during the first tick challenge study group, fed to repletion, dropped off, and molted normally to active adults. Despite evidence of the inflammatory responses described above, mice did not appear to recognize the presence of feeding ticks, did not exhibit irritation, itching, or scratching at the bite sites. Very few differences were noted in the intensity of the inflammatory response in the different mice used in the study. The one mouse examined at H2 had no inflammatory response. The only differences were among samples from mice examined on days 2 and 3; 4 samples from the mouse examined on day 2 showed a minimal (+) response, while the inflammation had progressed to mild (++) in 2 others; the one sample from the mouse examined on day 3 also showed mild (++) inflammation (Table 1, first challenge).

### *P. leucopus* Third Tick Challenge

Excluding the mice that were euthanized for sampling in the first tick challenge, tissue samples were collected from several of the same animals infested again during the third tick challenge.

*Peromyscus leucopus* tissues examined within 2 h (H2) post-challenge (third tick exposure) showed no evidence of an inflammatory response. However, 2 of 4 tissue samples examined within 6 h post third challenge (H6) show evidence of an initial inflammatory response; 3 of 4 tissue samples examined within 12 h post third-challenge (H12) showed a substantially more intense dermal inflammation than observed earlier, with mixed leukocytic infiltrates (Table 1, third challenge).

**Figure 6:**
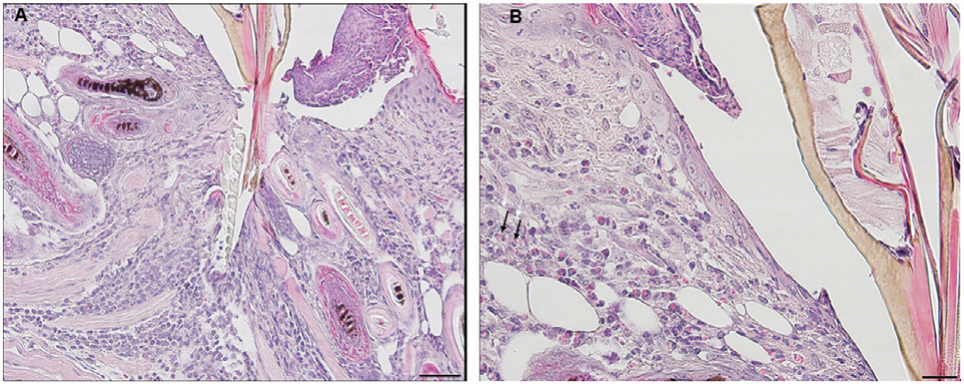
Photomicrographs showing histopathology of mouse (*Peromyscus leucopus*) skin from the ear collected on day 1 of third tick challenge (D1). Early onset of acute inflammatory response with infiltrating neutrophils and some eosinophils. Stain H&E. See text for details. **(A)** Low magnification image showing the vicinity of the bite lesion. The tissues show a severe focally diffuse subacute dermatitis. Bar = 100 µM. **(B)** Detail of tick bite lesion adjacent to tick mouthparts. The tissues show a severe focally diffuse subacute dermatitis with mixed neutrophilic, mastocytic, and histiocytic infiltrates but relatively few eosinophils (black arrows). There is focal epidermal hyperplasia with serocellular crusting. There is focal epidermal hyperplasia with serocellular crusting. Bar = 50 µM.

On day 1 following tick attachment in animals exposed for a third challenge the tissues near the tick-bite lesion show a severe focally diffuse subacute dermatitis with mixed leukocytic infiltrates, including some eosinophils (black arrows). There is focal epidermal hyperplasia with serocellular crusting. The lesion is sessile, without evidence of cavitation (Figures 6A,B).

**Figure 7:**
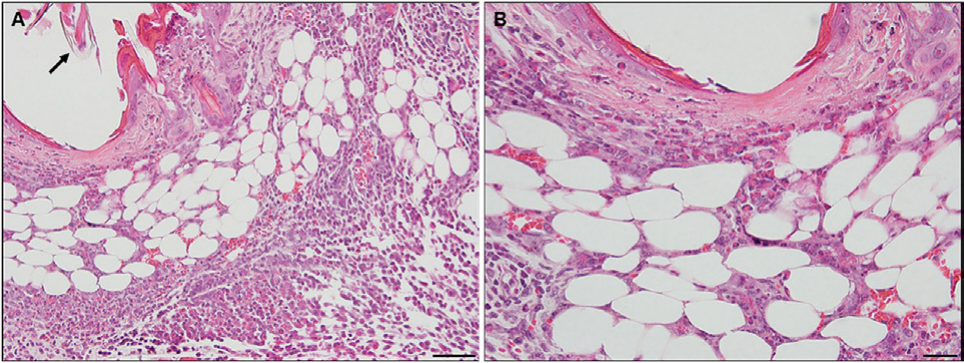
Photomicrographs showing histopathology of mouse (*Peromyscus leucopus*) skin from the ear collected on day 2 of third tick challenge (D2). The inflammatory response is more intense, widespread, with mixed leukocytic infiltrates, serocellular crusting, and dermal hyperplasia. The lesion appears sessile, without cavitation. Stain H&E. See text for details. **(A)** Low magnification image showing the tissues near the tick mouthparts (black arrow), forming a sessile lesion. There is a severe focally diffuse subacute dermatitis with numerous leukocytes. Black arrow indicates edge tick mouthparts. Bar = 100 µM. **(B)** Higher magnification images of area of the tick-bite lesion. In addition to the numerous infiltrating neutrophils and some eosinophils, there is increasing edema, along with focal muscle breakdown and other tissue necrosis. Bar = 50 µM.

By day 2, the inflammatory response is like that observed on day 1, but more intense with widespread mixed leukocytic infiltrates, serocellular crusting, and epidermal hyperplasia. There is increasing edema, along with focal muscle breakdown and other tissue necrosis. The lesion remains sessile, without cavitation. The infiltration of leukocytes into the tick-bite lesion is more intense than on the previous day (Figures 7A,B).

**Figure 8:**
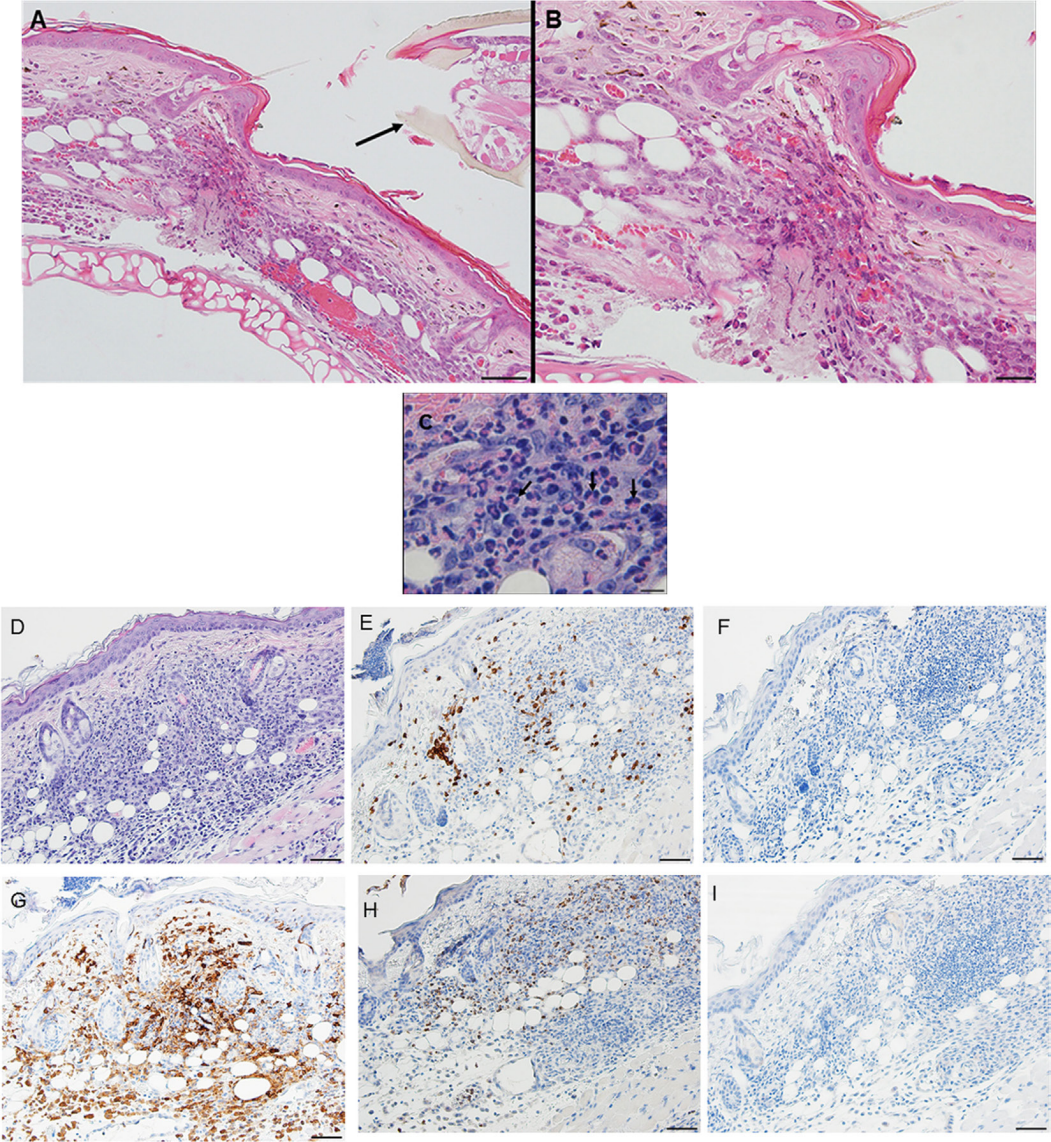
Photomicrographs showing high magnification image illustrating the histopathology of mouse (*Peromyscus leucopus*) skin on day 3 of the third tick challenge (D3). See text for details. **(A)** Dermal region adjacent to the tick mouthparts (black arrow) showing the intense, acute dermatitis forming an extensive sessile lesion, substantial leukocytic infiltrates, increasing hyperplasia, and hyperkeratosis. Bar = 100 µM. **(B)** Higher magnification images of area the tick attachment site, showing the focal lesion with extravasated red blood cells and leukocytic infiltrates. Bar = 50 µM. **(C)** Higher magnification image of same region showing representative eosinophils (black arrows). Bar = 20 µM. **(D–I)** Photomicrographs showing high magnification images illustrating the histopathology of mouse (*Peromyscus leucopus*) skin on day 3 of the third tick challenge (D3). **(D)** Section of mouse skin selected stained with H&E for comparison with immunohistochemical (IHC) markers. Bar = 50 µM. **(E–I)** IHC markers (brown stain) for different leukocyte cell types, in or near the tick-bite lesion (box). **(E)** CD3 identifying T-lymphocytes, dispersed within the focal area of the tick-bite lesion. **(F)** Eosinophil major basic protein for eosinophils; very few eosinophils were detected in or near the tick-bite lesion. **(G)** IBA1 identifying numerous macrophages in the tick-bite lesion and surrounding dermal tissues. **(H)** Myeloperoxidase showing numerous neutrophils in the tick-bite lesion. **(I)** Mmcp-8 for basophils; no basophils were found. Bars = 50 µM.

By day 3, the response of the host tissues is like that seen on the previous day, but now also shows blood vessel congestion with leukocytes marginating within the vessels. Moreover, the severity of the inflammation within the area of the lesion is more widespread and sessile (Figures 8A–C). High magnification images of the dermal tissues surrounding the bite lesion shows numerous invading leukocytes, including a few eosinophils, with their characteristic bilobed nuclei and eosin-staining cytoplasm (Figure 8C, black arrows). IHC staining shows that the mixed leukocytic infiltrate is primarily comprised of T-cells and macrophages, with occasional neutrophils, rare eosinophils, but no basophils (Figures 8E–I). An H&E image from the identical skin section is included (Figure 8D) to facilitate comparisons with the IHC-stained sections.

**Figure 9:**
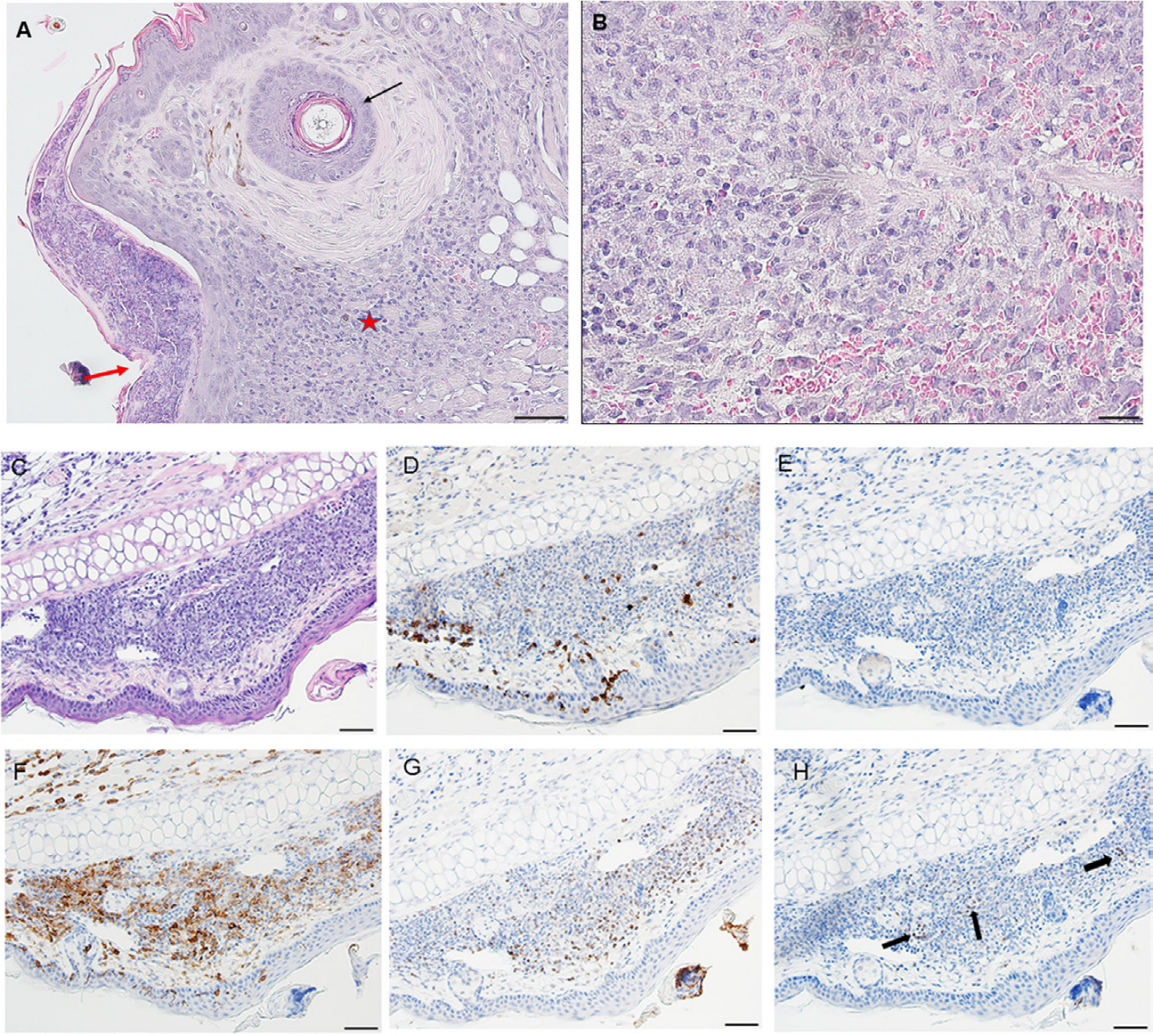
Photomicrographs showing high magnification image illustrating the histopathology of mouse (*Peromyscus leucopus*) skin on day 4 of the third tick challenge (D4). The skin near the tick-bite shows an extensive, predominantly granulomatous mixed leukocytic dermatitis. Stain H&E in **(A,B)**. **(A)** Low magnification image shows the tick-bite lesion filled with masses of mixed leukocytic cells, extensive hyperplasia, and serocellular crusting. Black arrow indicates hair follicle; red asterisk denotes intense granulomatous area. Red arrow indicates vicinity of tick bite. The lesion is sessile, no cavity formation evident. Bar = 100 µM. **(B)** Higher magnification image of the tick-bite lesion. There are many macrophages and abundant histiocytes interspersed with numerous neutrophils and occasional eosinophils. There is widespread hemorrhaging, diluted and ruptured blood vessels, and tissue necrosis. Bar = 50 µM. **(C–H)** Photomicrographs illustrating the histopathology of mouse (*Peromyscus leucopus*) skin on day 4 of the third tick challenge (D4). The skin near the tick-bite shows an extensive, predominantly granulomatous mixed leukocytic dermatitis. **(C)** Section of mouse skin selected stained with H&E for comparison with immunohistochemical (IHC) markers (box). Bar = 50 µM. **(D–H)** IHC markers (brown stain) for different leukocyte cell types, in or near the tick-bite lesion. **(D)** CD3 identifying T-lymphocytes, dispersed within the focal area of the tick-bite lesion. **(E)** Eosinophil major basic protein for eosinophils; very few eosinophils were detected in or near the tick-bite lesion. **(F)** IBA1 identifying numerous macrophages in the tick-bite lesion and surrounding dermal tissues. **(G)** Myeloperoxidase showing numerous neutrophils in the tick-bite lesion. **(H)** mMCP-8 for basophils. Arrows indicate few, scattered basophils, mostly in small blood vessels. Bars = 50 µM. See Figure 10 for a higher magnification of the IHC assay.

**Figure 10:**
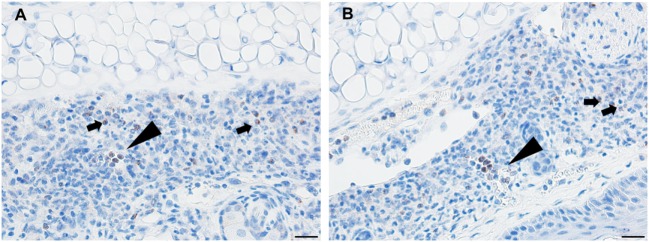
Photomicrographs illustrating the results of an immunohistochemical (IHC) assay for basophils in mouse (*Peromyscus leucopus*) skin on day 4 of the third tick challenge (D4). **(A,B)** Two separate sections of tick-bite lesion showing basophils (arrows). Most of the IHC-positive cells occur within the lumens of small blood vessels (triangular arrows); a few are scattered in the dermis (small arrows). Bar = 20 µM.

By day 4, the condition has progressed to a predominantly granulomatous dermatitis, focally severe with extensive epidermal hyperplasia and serocellular crusting. Epithelioid macrophages have infiltrated and are interspersed with numerous neutrophils and few eosinophils (as determined by IHC). There is widespread hemorrhage, diluted and ruptured blood vessels, and tissue necrosis (Figures 9A,B). IHC staining shows that the mixed leukocytic infiltrate is still primarily comprised of T-cells and macrophages, in addition to many neutrophils dispersed throughout the broad, sessile lesion. There were very few eosinophils and occasional basophils (black arrows) (Figures 9C–H). Figures 10A,B is a higher magnification IHC image showing that while rare, most of the basophils found were in the lumen of small blood vessels. An H&E image from the identical skin section is included (Figure 9C) to facilitate comparisons with the IHC-stained sections.

**Figure 11:**
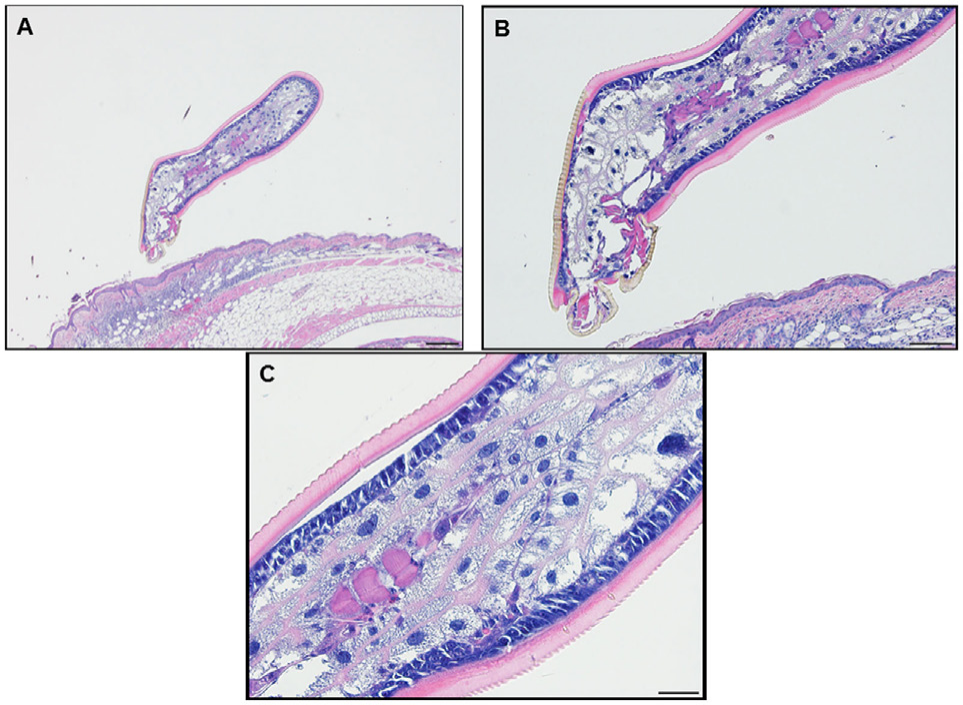
Photomicrographs showing a feeding *Ixodes scapularis* nymphs attached to skin of a mouse (*Peromyscus leucopus*) on day 3 of the third tick challenge (D3) showing the normal internal body tissues. Stain H&E. **(A)** Low magnification image showing the entire tick body. Bar = 100 µM. **(B)** Higher magnification image showing the tick’s anterior body region. Bar = 50 µM. **(C)** High magnification image showing the interior of the tick’s body tissues. The midgut epithelial cells have expanded greatly with masses of hematin accumulating after hemoglobin digestion. The epidermis is enlarged, indicating cuticle growth consistent with the blood engorging tick body. Bar = 20 µM.

Feeding ticks examined during the third tick challenge did not show evidence of damage to their midgut epithelial cells or other internal tissues. Despite very strong inflammatory responses in the region of the tissue surrounding the tick bites, the *I. scapularis* nymphs could engorge normally (Figure 11). Also noteworthy was that none of the mice showed evidence of irritability, loss of appetite, or any other visible indications of distress due to multiple tick feeding.

Compared to the first infestation, considerable differences were noted in the intensity of the inflammatory response in the different tissue samples from the mice exposed to ticks during the third infestation. Differences were noted at H6, 3 minimal (+), 3 none; at H12, 1 mild (++), 1 moderate (+++), and 1 none; on D2, 1 minimal (+), 3 mild (+ +), 4 that were moderate (+++), and 1 very strong (++++); on D3, 1 minimal (+), 2 mild (++), 2 moderate (+++); on day D4, 2 moderate (+++) and 4 very strong (++++) (Table 1, third infestation).

### *C. porcellus* First Tick Challenge

Examination of skin tissues on days 1 and 2 post-challenge (first tick exposure) showed no evidence of an inflammatory response.

**Figure 12:**
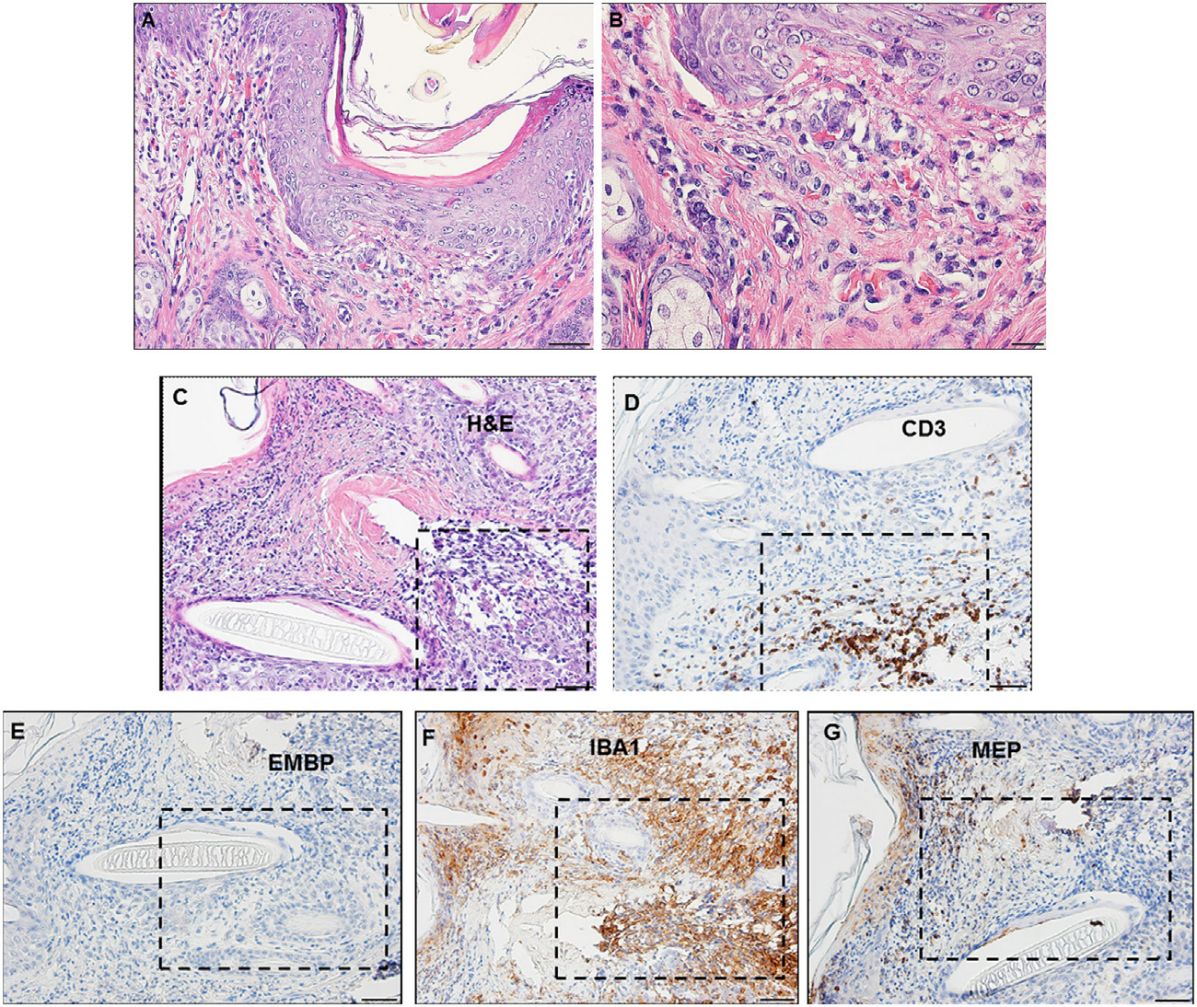
Photomicrographs showing the histopathology of guinea pig (*Cavia porcellus*) skin on day 3 of first tick challenge (D3) near the tick mouthparts. There is hyperplasia and keratosis in the epidermal layer next to the tick’s mouthparts. There is leukocytic infiltration indicating an early inflammatory response. Stain H&E. **(A)** Low magnification image showing the skin region adjacent to the tick mouthparts. Bar = 100 µM. **(B)** High magnification image of the skin tissues next to the tick mouthparts. Bar = 50 µM. **(C)** Section of guinea pig skin stained with H&E for comparison with Immunohistochemical (IHC) markers (box). Section shows tissue necrosis and cavitation in the focal area of the tick-bite lesion. Bar = 50 µM. **(D–G)** IHC markers (brown stain) for different leukocyte cell types, in or near the tick-bite lesion. **(E)** CD3 identifying T-lymphocytes, concentrated around margins of the tick-bite lesion. **(F)** Eosinophil major basic protein for eosinophils; none were detected in or near the tick-bite lesion. **(G)** IBA1 identifying numerous macrophages throughout the tick-bite lesion and surrounding dermal tissues. **(H)** Myeloperoxidase showing numerous heterophils in the tick-bite lesion. Bars = 50 µM.

By days 3 and 4 post-challenge, the host skin revealed a localized epidermal hyperplasia and hyperkeratosis in the area immediately adjacent to the tick’s mouthparts. Examination of the H&E images (Figures 12A,B) indicates a mixed leukocytic infiltration and extensive tissue necrosis, leading to the development of a cavitary lesion. IHC staining shows a mixed leukocytic infiltrate comprised mostly of macrophages and heterophils (i.e., neutrophil-like leukocytes common to guinea pigs) throughout the lesion, T-cells but eosinophils were minimal to absent. T-cells were concentrated along the margins of the lesion (Figures 12C–G). The ticks remained attached and appeared to have fed normally, despite the hosts’ inflammatory response.

### *C. porcellus* Third Tick Challenge

**Figure 13:**
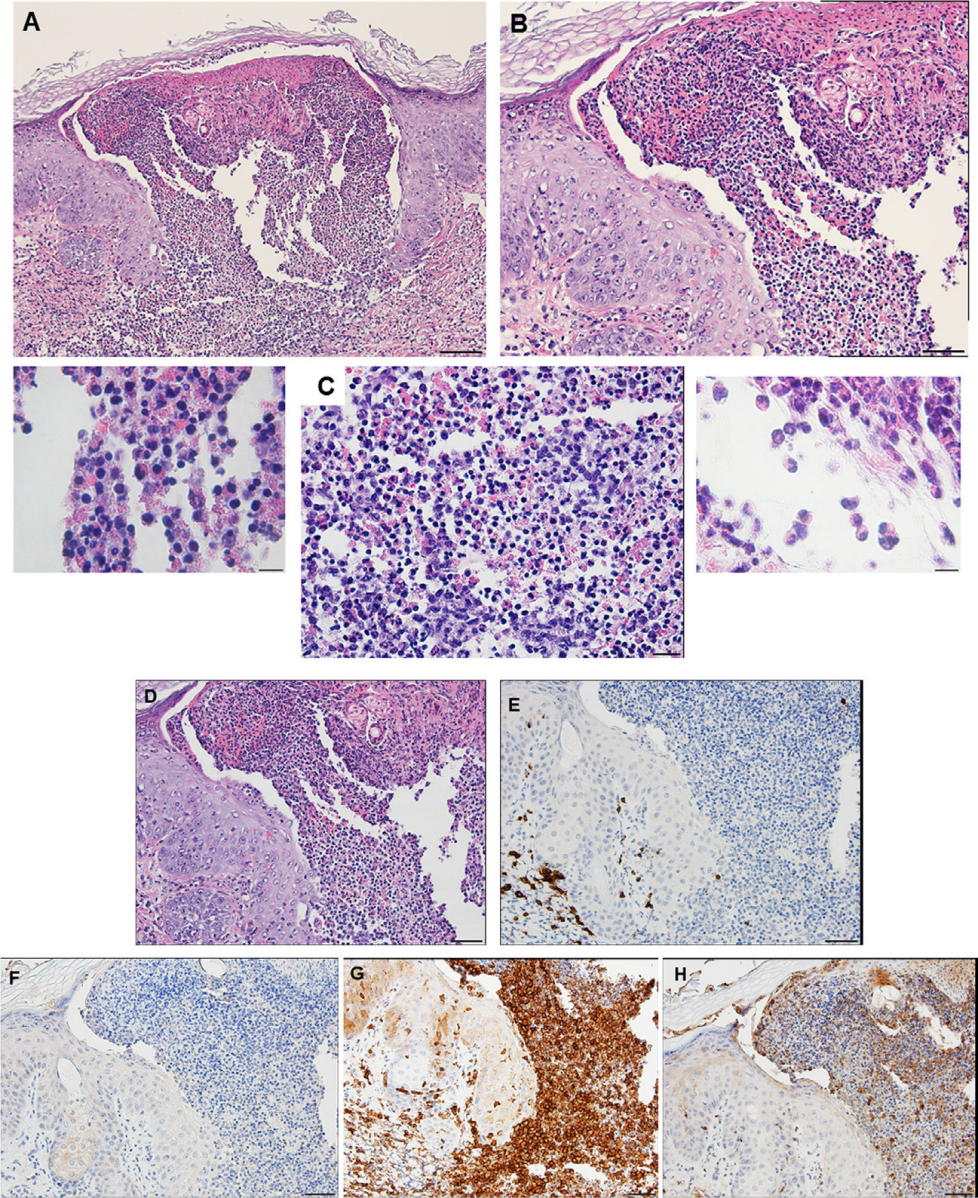
Photomicrographs showing the histopathology of guinea pig (*Cavia porcellus*) skin on day 3 of third tick challenge (D3) near the tick bite. Stain H&E. See text for details. **(A)** Low magnification image showing the tissues adjacent to the tick-bite. The section shows a severe, focally diffuse inflammation with numerous neutrophils, eosinophils, macrophages, and mononuclear cells, also with a dense serocellular crust, very severe epidermal hyperplasia and hyperkeratosis. There is a very large cavity lesion in the center. Bar = 100 µM. **(B)** High magnification image showing region of the tick-bite lesion, with masses of mixed, infiltrating leukocytes, severe epidermal hyperplasia, hyperkeratosis, a broad cavity lesion, breakdown of dermal collagen, and extensive hemorrhage. Bar = 50 µM. **(C)** Very high magnification image showing masses of neutrophils and eosinophils in the tick-bite lesion, especially concentrated in the central cavity. Insets either side of image C indicate eosin-staining degranulating leukocytes, presumably eosinophils. Bar = 20 µM. **(D–H)** Immunohistochemical markers (brown stain) for different leukocyte cell types, in or near the tick-bite lesion. **(E)** CD3 identifying T-lymphocytes, concentrated around margins of the tick-bite lesion. **(F)** Eosinophil major basic protein for eosinophils; few eosinophils were recognized by this stain in or near the tick-bite lesion. **(G)** IBA1 identifying numerous macrophages throughout the tick-bite lesion and surrounding dermal tissues. **(H)** Myeloperoxidase showing numerous neutrophils in the tick-bite lesion. The tick had fallen off. Bars = 50 µM.

Compared to the first tick challenge, examination of skin sections showed that there was an intense inflammation beginning as early as day 1 post-challenge (third tick exposure). On day 2 post third tick challenge, the tissues showed a severe, focally diffuse, mixed superficial to mid-dermal inflammatory infiltrate composed mostly of very numerous heterophils and macrophages, but also including occasional mast cells, histiocytes (=tissue macrophage), and mononuclear cells but few or no eosinophils. There was a very dense serocellular crust, severe epidermal hyperplasia, and hyperkeratosis surrounding the site of tick attachment. There was substantial tissue damage surrounding the bite lesion, which had expanded to a broad cavitary lesion, which likely resulted from extensive loss of dermal collagen. Overall, the presentation was that of an extremely intense mixed neutrophilic dermatitis, with numerous degranulating polymorphonuclear leukocytes (figure not shown). By days 3 and 4 post-third challenge, the presentation was like that seen on the previous days but the leukocytic infiltrate was even more intense and widespread, covering the entire width of the tissue sample. The tissue sample revealed an intense granulomatous mixed macrophage–neutrophilic dermatitis with substantial hemorrhage. The cavity lesion noted on day 2 post-challenge was larger and filled with masses of leukocytes, most of which were eosin-stained cells and granules. Much of the hyperkeratosis had intensified to form scab-like structure, which had begun to break loose (Figures 13A–C). IHC staining revealed an intense macrophage and heterophil dominated inflammation, with T-cells around the margins but very few eosinophils (Figures 13D–H). Basophils could not be confirmed since the mouse-specific mMCP-8 did not work in the guinea pig samples. Most ticks that attached during the third tick challenge either failed to feed or died while attached (Figures 13D–H). The tick-sensitized guinea pigs showed elevated levels of irritability, with numerous scratch marks on the skin, bleeding, and an erythematous appearance, from attempts to dislodge ticks, which they could not remove because of the Elizabethan collar.

**Figure 14:**
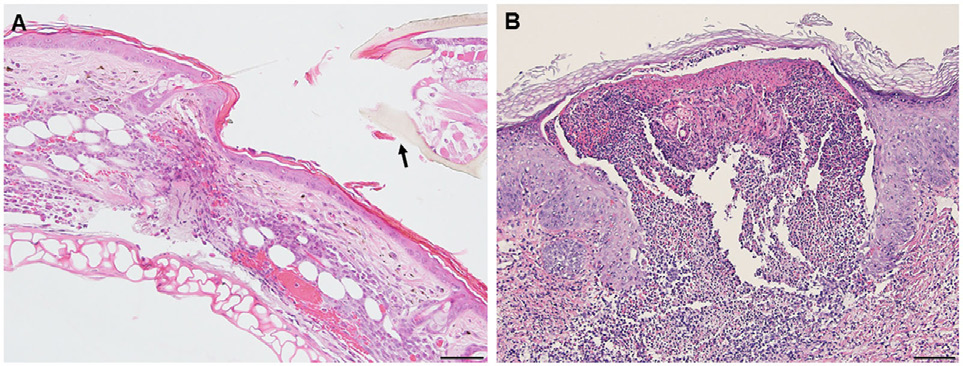
Photomicrographs comparing the histopathology of the mouse (*Peromyscus leucopus*) versus guinea pig (*Cavia porcellus*) tick-bite dermal lesion on day 3 of the third tick challenge. Stain H&E. **(A)** Mouse dermal region adjacent to the tick mouthparts (black arrow) showing an acute dermatitis forming a broad sessile lesion, substantial leukocytic infiltrates hyperplasia, and keratosis. Arrow indicates tick. Bar = 100 µM. **(B)** Guinea pig dermal lesion showing an acute dermatitis forming a large focal cavity lesion, with numerous heterophils, macrophages, histiocytes and mononuclear cells, dense serocellular crusting, severe hyperplasia, and hyperkeratosis. The scab-like keratotic layer has separated from the underlying dermis. Tick had fallen off. Bar = 100 µM.

A comparison of the inflammatory responses to *I. scapularis* tick bites in the two different hosts is shown in Figure 14.

## Discussion

The results of this study show that *I. scapularis* nymphs could engorge repeatedly on *P. leucopus* despite an increasingly intense dermal inflammation. This finding suggests that the host immune incompetence hypothesis (15) does not fully explain why these wild mice failed to prevent further tick feeding. Even though their tissues were inflamed, the animals did not exhibit signs of irritability, scratching, or itching and no attempts to remove the feeding ticks. The tick clearly did not prevent the presence of a mouse cellular infiltrate, perhaps because its saliva compromised the agonists released by these inflammatory cells. Thus, the “immune evasion” hypothesis could be reframed as the “immune agonist evasion” hypothesis. Our findings reveal the fundamental similarity of the *P. leucopus* inflammatory response with that of previous findings reported for laboratory mouse models, but not the natural sylvatic host for this tick species [e.g., Ref. (12, 17)]. However, in *P. leucopus*, the tick-bite lesion was sessile, i.e., the dermal structure was not substantially disrupted, an immune response feature not previously reported. Guinea pigs, however, subjected to repeated tick bites responded as described in previous reports (18, 19), i.e., with an intense and predominantly histiocytic dermal inflammatory response. They also developed a large cavitary lesion filled mostly with leukocytes and a prominent scab-like epidermal hyperkeratosis weakening the tick’s attachment, along with irritability, scratching, and itching behavior, all of which led to tick rejection. So far, as the authors can determine, the contrasts in dermal lesion architecture between the two different host species has not been previously reported and may further highlight a novel factor contributing to the tick’s ability to feed successfully.

We suggest that the reason for the difference in host response to repeated tick challenge may not be solely due to the types of immune cells that infiltrated the wound sites, since both animals presented with a relatively similar immune cell profile. Rather, the difference appears to be mainly due to the differences in the lesion architecture; sessile with little dermal disruption and only mild hyperplasia/hyperkeratosis in white footed mice versus a focal, cavitary lesion with substantial disruption of the dermal tissues and severe epidermal hyperplasia/hyperkeratosis in the guinea pigs (Figure 14). It may also have been due to how the two different animals interacted at the molecular level in response to immunogenic proteins in tick saliva (3, 19) and their effects on their host tissues. Our findings for *P. leucopus* are generally consistent with the findings for laboratory mice (BALB/c) also subjected to *I. scapularis* reinfestations (12) except for some important differences. Those authors observed very few lymphocytes, neutrophils, or eosinophils during the first tick challenge, but a substantial increase in these same inflammatory cells during a second tick challenge. Especially noteworthy is that they observed as many eosinophils as there were neutrophils, as well as lymphocytes and some macrophages. In contrast, the response we observed in tick-sensitized *P. leucopus* showed very few eosinophils but an intense infiltration and concentration of macrophages, T-lymphocytes, many neutrophils, and a few basophils, perhaps because the previous authors only used H&E staining whereas we used both H&E and IHC analysis to further characterize cellular infiltrates.

If the immune cell infiltrate comprising the dermal inflammation is similar in the two different animals, what accounts for the different histopathological presentation and its effect on tick feeding, namely, tolerance in *P. leucopus* versus rejection in *C. porcellus*? Evidence from previously published studies that might explain the significance of the different histopathologic presentations is described below.

A substantial amount of literature concerning the histopathology of host dermal tissues in response to tick bites has accumulated over the past several decades. The limited dermal inflammation seen in tick-naïve animals versus the more intense acute inflammation with numerous neutrophils, macrophages, eosinophils, basophils, and other immune cells seen in response to repeated tick challenges is a familiar story (4). Studies done with wood ticks (*Dermacentor andersoni*) feeding on guinea pigs showed that among the immune cell types noted previously, basophils also infiltrated in large numbers and degranulated adjacent to the feeding lesion (15). Basophils are a major source of histamine and histamine was significantly greater in the tick-resistant than in tick-naïve animals. Histamine release has been reported to induce reduced tick sucking and salivation (20).

The role of basophil-mediated histamine transport to tick feeding sites has received relatively little attention in mice. Nicholson et al. (1) reported that “histamine-induced edema does not occur in the white-footed mouse.” However, in laboratory mice subjected to repeated tick feeding by *Haemaphysalis longicornis*, basophils were found to be essential for antibody-mediated acquired immunity. Acquired resistance to ticks was lost when basophils were ablated (21). Basophils were reported to occur in the blood of *P. leucopus* and other mouse species (22), and we confirmed their presence (by IHC) in the tissues from tick-sensitized *P. leucopus*. However, few were found and then mostly within the lumens of small blood vessels, suggesting that basophils had little if any role in the anti-tick inflammatory response. We were unable to compare basophil occurrence in tick-sensitized guinea pigs since the IHC assay was mouse specific. However, basophil-mediated hypersensitivity responses in ixodid tick-sensitized guinea pigs is well known (4). Similar findings were reported for guinea pigs infested with soft ticks, where basophils represented 48–56% of all immune cells (23).

The tick-bite lesion is believed to be mostly the result of macrophage and neutrophil activity, likely initiated by early infiltration of T-cells that secrete proinflammatory cytokines. Invading neutrophils degranulate and release enzymes, especially serine proteases (24) and MPO (25), enzymes that damage tissues, thereby expanding the bite lesion as a feeding pool from which the ticks suck blood. Early invading macrophages, M1 type (26), can secrete proinflammatory cytokines, nitric oxide (NO), and reactive oxygen species (ROS) (26, 27). In laboratory mice (BALB/c, *Mus musculus*), tick feeding (*D. andersoni*) was found to upregulate chemokines, cytokines, and other chemoattractants that attracted neutrophils and monocytes to the dermal lesion as well as keratinocytes. In addition to the tissue breakdown described earlier, the chemotaxis also led to hyperkeratinization, dermal hyperplasia, and oxidative stress involving ROS (17). Similar findings regarding neutrophil and macrophage chemoattractants were reported in humans bitten by ticks, *Ixodes ricinus* (28). Whether basophils, known to secrete histamine and other cytotoxic molecules (29) also contribute to the inflammatory response in *P. leucopus* seems unlikely in view of the small numbers found and mostly confined to blood vessels.

In African cattle, the predominant cells infiltrating tick attachment sites in highly resistant animals were eosinophils while the predominant cells in poorly or non-resistant animals were neutrophils (30). In cattle exposed to cattle ticks, *Rhipicephalus microplus*, the highly tick resistant “indicus” breed had significantly more basophils and eosinophils at the bite sites compared with tick-susceptible “taurus” breed (31). Sheep (*Ovis aries*) infested with *I. ricinus* ticks developed a strong initial inflammatory response dominated by neutrophils. Following the tertiary infestation, when the animals became resistant, the inflammation was characterized by increasing numbers of eosinophils and rapid degranulation by mast cells and basophils (32).

Further studies, especially further molecular studies, are needed to explain the different host responses to repeated tick bite challenge. For example, *I. scapularis* could evade their hosts’ alternate complement pathway since it expresses *ISAC* (33), which acts against host complement in *P. leucopus*, thereby disabling one arm of the immune response, which was implicated in tick rejection reactions (34, 35). However, it is not known whether these same antagonistic proteins and peptides are also effective against similar host targets in a very different host such as the guinea pig. Perhaps these tick salivary compounds have co-evolved with immune peptides in their primary host, the white-footed mouse?

## Ethics Statement

This study was carried out in accordance with the recommendations in the Guide for the Care and Use of Laboratory Animals of the National Institute of Health. The protocol was approved by the National Institutes of Health Animal Care and Use Committee protocol (ASP LMVR6).

## Author Contributions

JA infested white-footed mice with ticks, *I. scapularis*, examined responses, collected and processed tissue samples, reviewed, edited and contributed to figures and final manuscript draft. DS evaluated tissue slides for inflammation, helped prepare images, wrote preliminary manuscript, prepared figures, wrote and edited the final draft. JV reviewed tissue images, helped prepare manuscript, reviewed and edited final draft. JR reviewed tissue images, helped prepare manuscript, reviewed and edited final draft. BMN carried out the IHC assays on the host tissues. IM evaluated and described inflammatory responses, prepared histopathological and immunohistochemical images, helped prepare manuscript, reviewed and edited final draft.

## Conflict of Interest Statement

The authors declare that the research was conducted in the absence of any commercial or financial relationships that could be construed as a potential conflict of interest.
